# Microbial Diversity of the Surface of Polypropylene and Low Density Polyethylene‐Based Materials (Plastisphere) From an Area Subjected to Intensive Agriculture

**DOI:** 10.1002/mbo3.70121

**Published:** 2025-12-08

**Authors:** Diego Becerra, Gema Rodríguez‐Caballero, Frutos Carlos Marhuenda‐Egea, Alfonso Olaya‐Abril, Conrado Moreno‐Vivián, Lara Paloma Sáez, Victor Manuel Luque‐Almagro, María Dolores Roldán

**Affiliations:** ^1^ Departamento de Bioquímica y Biología Molecular, Edificio Severo Ochoa, Campus de Rabanales Universidad de Córdoba Córdoba Spain; ^2^ Departamento de Agroquímica y Bioquímica Instituto Multidisciplinar para el Estudio del Medio Ramón Margalef, San Vicente Del Raspeig Alicante Spain

**Keywords:** agriculture, plastisphere, polyethylene, polypropylene, synthetic plastic

## Abstract

Accumulation of synthetic plastics in the biosphere has led to global pollution, provoking serious consequences for the environment and human health. Uncontrolled agricultural plastic landfills have the risk of becoming a source of agrochemicals and microplastics. Biotechnological approaches to solve plastic pollution include the removal of these polymers through biological degradation, which is a friendly environmental method. The microbial communities colonizing plastic debris (plastisphere) are considered as a potential source of plastic‐degrading microorganisms. In this study, a bacterial biodiversity analysis, based on 16S rRNA gene‐targeted metagenomic sequencing, was achieved in the plastisphere of low‐density polyethylene (LDPE) and polypropylene (PP) polymers from an agricultural landfill. The α‐diversity analysis did not show significant differences between LDPE and PP plastispheres and the plastic‐free bulk soil, while LDPE and PP bacterial communities clustered close, but separately from the bulk soil in a β‐diversity analysis. Although the taxonomic composition of both plastispheres was different, they shared a significantly higher proportion of Cyanobacteria and Deinococcota than the bulk soil. Additional analyses showed different indicator families, genera and species that can be associated with plastispheres. A predictive functional analysis suggests that degradation of plastic additives in both plastispheres is probably occurring. In addition, the existence of degradation processes for specific herbicides in each plastisphere is highlighted, and the possible exposure of LDPE to both physical and biological degradation processes is also described. These results will contribute to characterize the soil plastisphere exposed to different environmental conditions, and to understand the specific biological niches where plastic‐degrading microorganisms could survive.

## Graphical abstract

Bacterial communities colonizing LDPE and PP plastispheres from an intensive agriculture landfill exhibited differences in their taxonomic composition and structure. A predictive metagenomic analysis suggested physical and biological degradation of LDPE, in addition to degradation of plastic additives and herbicides by microorganisms present in both plastispheres.

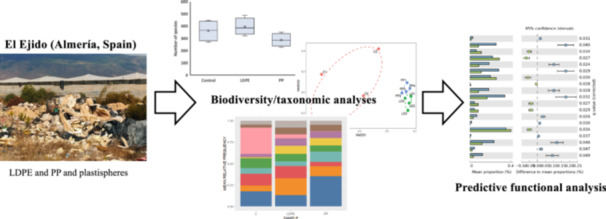

## 1 Introduction

The extensive use of fuel‐derived plastics in modern society and the recalcitrant nature of these polymers are two key factors that contribute to the massive accumulation of synthetic plastics around the world. According to the United Nations Environment Programme, over 460 million metric tons of synthetic plastics are produced every year. Polymers composing these synthetic plastics display different chemical natures, which determine their mechanical properties (strength, stiffness, elasticity, etc). Polyethylene (PE) and polypropylene (PP) are the most abundant oil‐based plastics, but polystyrene (PS), polyethylene terephthalate (PET), and polyvinyl chloride (PVC) are also frequently found in plastic waste (Moharir and Kumar 2019). Plastic inputs are mainly land‐based, and they include urban littering, packaging, different industrial activities, and agriculture, among others. In agriculture, plastic‐mulching is widely used around the world. Plastic mulch, which is composed mainly of highly resistant low‐density polyethylene (LDPE), is used in agriculture to increase soil temperature and to minimize water evaporation. Other plastics used in agriculture include greenhouse coverings, irrigation tubes, silage, and pesticide containers. These products are composed mainly of PP, PVC, and linear low‐density polyethylene (LLDPE). The low value of most agricultural plastics, the cost of transport and recycling, and their contamination with pesticides or fertilizers often lead to their illegal dumping. These uncontrolled plastic landfills have the risk of becoming a source of agrochemicals and microplastics (plastic particles < 5 mm in size) leaking into soil, water, and air in areas that are very productive in food for human consumption (Petersen and Hubbart 2021). Thus, a more sustainable use of plastics in agriculture is one of the current environmental challenges (Hofmann et al. 2023). The accumulation of synthetic plastics in the biosphere has led to global pollution, provoking serious consequences for the environment and living beings. Plastics directly damage sea life, and polymer derivatives like PET may act as endocrine disruptors (Wagner and Oehlmann 2009).

On the other hand, degradation of these petroleum‐derived plastics by physico‐chemical factors generates micro/nanoplastics, which enter the food chain, interact with other environmental pollutants and pathogens, and alter the biophysical properties of soil, resulting in considerable ecotoxicological effects (De Souza Machado et al. 2018; Maity et al. 2021; Zhang et al. 2024). The protection and safeguarding of the environment and citizens from the enormous burden of plastic waste is one of the main policies framed under the “European Strategy for Plastics in a Circular Economy” (European Commission 2018). Although there are physical‐chemical treatments for the elimination of synthetic plastics, they have major drawbacks. Thus, incineration of plastic requires high energy consumption, and dangerous gaseous compounds are produced, while chemical treatments are limited to small‐scale operation (Moharir and Kumar 2019).

Taking advantage of the fact that plastics are a suitable source of carbon and energy for bacteria, biological degradation of these polymers is considered a friendly environmental alternative. However, these synthetic plastics present a main drawback of bioaccessibility, and their biodegradative processes depend on the chemical structure of each polymer (hydrophobicity, molecular weight, and crystallinity), being strongly influenced by environmental conditions. Synthetic plastics like PE, PS, PP, and PVC are characterized by their chemical inertness, and they are very resistant to biodegradation. By contrast, PET polymer is more accessible to microorganisms because it presents a low degree of crystallinity. During the biodegradation of plastics, extracellular enzymes catalyze the polymer fragmentation, and the products and monomers generated are further assimilated by bacteria (Crystal Thew et al. 2024). Diverse bacteria have been identified with the ability to degrade PET, such as the strain *Ideonella sakaiensis* 201‐F6. This bacterium presents the enzymes polyethylene terephthalate hydrolase (PETase) and mono(2‐hydroxyethyl) terephthalic acid hydrolase (MHETase), which enable the utilization of PET as a carbon source (Yoshida et al. 2016). Recently, a glutathione peroxidase involved in depolymerization of LDPE has been described as part of a multienzyme pathway for LDPE biodegradation in *Rhodococcus* sp. C‐2 (Rong et al. 2024). This novel bacterial strain exhibited an unusually high efficiency in LDPE degradation, in contrast to other bacterial strains as stated by the limited studies about the biodegradation of this and other recalcitrant plastics. On the other hand, polypropylene‐degrading bacteria have been reported, such as several *Bacillus* strains able to detoxify blends of PP and poly‐l‐lactide (PLLA) in minimal media as well as in soil (Jain et al. 2018). In addition, bacteria from the gut microbiota of mealworms have been described to degrade PP, among other plastics (Yang et al. 2021).

An alternative to the use of petroleum‐derived plastics could be based on the application of polymers derived from crop biomass (bioplastics) that reduce carbon dioxide emissions during their biosynthesis, but it does not guarantee their biodegradation. Only the bio‐based and biodegradable plastics, such as thermoplastic starch (TPS), polyhydroxyalkanoate (PHA), and polylactic acid (PLA), represent a suitable alternative to the synthetic and nonbiodegradable plastics, but their elevated production cost is a factor against their extensive use (Narancic and O'Connor 2019). Hence, one of the main issues regarding plastic pollution is focused on searching for microorganisms with the ability to degrade the highly recalcitrant PE, PS, and PP polymers that significantly contribute to environmental pollution.

The microbial community colonizing plastic debris, defined as plastisphere, is considered a suitable source of plastic‐degrading microorganisms (Singh et al. 2024). Plastisphere has been broadly described for plastic debris deposited in water. By contrast, the soil plastisphere, concerning plastic residues on soil, has received less attention, even though in recent years various studies reveal its great relevance in soil communities (Chae and An 2018; Rillig et al. 2024). The study of soil plastisphere has been carried out by several approaches, including field collection of plastic material, incubation of plastic materials in different natural environments, and mesocosm‐based approaches. In contrast to traditional molecular techniques, the application of next‐generation sequencing to metagenomics is providing a more complete and real knowledge about microbial diversity inhabiting the plastisphere (Amaral‐Zettler et al. 2020; Tiwari et al. 2022). According to previous studies, the microbial community of the plastisphere generally presents lower diversity than the nearby soil (bulk soil), finding different microbial taxonomic groups in the plastisphere compared to the surrounding soil (Rillig et al. 2024). Although microbial biodiversity from plastics deposited in different ecosystems is being elucidated, additional studies are required to establish a hypothetical plastisphere core microbiome. In addition to analyzing the microbial profile by targeted metagenomics, shotgun metagenomics allows the identification of genes present in the plastisphere microbiome (Saleem et al. 2023). Downstream multi‐omics techniques, including metatranscriptomics, metaproteomics, and metabolomics, may provide the functional analysis required for the understanding of gene expression profiles, metabolic functions, and regulation mechanisms that are predominant in ecosystems like the plastisphere, as well as for seeking out new enzymes with elevated potential for plastic biodegradation (Tiwari et al. 2022).

In the last decade, many studies about microbial biodiversity have focused on agricultural soils polluted with plastics as a consequence of mulching practice. However, the microbiome present in the plastisphere of agricultural plastic landfills has received less attention, despite it could constitute not only a source of plastic‐degrading microorganisms, but also a reservoir of microorganisms that might be resistant and/or display the capability to degrade other pollutants like pesticides or fertilizers (Chung et al. 2022). To facilitate the biodegradation of the plastic waste generated in agricultural practices, the effect of abiotic treatments of these residues has been described (Blesa Marco et al. 2024). In this study, the taxonomic profile of agricultural plastic landfill from an area of intensive agriculture located at the South‐East of Spain (El Ejido, Almería) has been studied using 16S rRNA gene‐targeted metagenomic sequencing. In addition, the functional characterization of the microbiome present in the agricultural plastisphere has been estimated by searching for associations between specific metabolic pathways and the chemical structure of the plastic waste analyzed. The results obtained in this study aim to provide insight into the overall picture of LDPE and PP plastispheres, and to understand the selective enrichment of microbial taxa in soils contaminated with these synthetic plastics, considering the potential influence of herbicide and pesticide residues. The identification of novel bacterial metabolic pathways potentially involved in the detoxification of LDPE and PP may contribute to the development of a sustainable modern agriculture.

## 2 Material and Methods

### 2.1 Sampling of Plastic Material

Plastic material used in this study was collected in October 2023 from an agricultural landfill site in El Ejido (Almería, Spain) (Figure S1). Four samples of two different plastic waste films based on PP and LDPE, which were half buried in the soil, were taken and transported to the laboratory in sterile bags. Four additional samples from nearby soil (1 m radius from the plastic materials) that were not directly in contact with plastics were also collected to be used as the plastic‐free control (bulk soil).

### 2.2 FTIR Analysis

To determine the molecular composition of plastics used in this study, three samples of each plastic were analyzed by Attenuated Total Reflection Fourier Transform‐Infrared Spectroscopy (ATR‐FTIR) using a BRUKER IFS 66. Spectra of individual samples were obtained with a resolution of 4 cm^−^^1^ and a scanning range from 4000 cm^−1^ to 600 cm^−1^. FTIR spectra showed the average of twenty scans, which were analyzed with the software MATLAB version 9.13.0 (The MathWorks 2022).

### 2.3 DNA Extraction and Purification

Plastisphere samples (250 mg) containing microorganisms adhered to the plastic directly or indirectly through soil particles attached to the plastic were collected by washing the plastic film with 50 mM Tris‐HCl (pH 7,5) buffer, and the suspensions were vortexed for 10 min and centrifuged at 5000 × *g* for 5 min. Pellets containing the cells were used for total genomic DNA extraction with the DNeasy PowerSoil Pro Kit (QIAGEN). In the case of the samples used as plastic‐free controls, the same amount of soil (250 mg) was used for DNA extraction. All samples were subjected to disruption using the FastPrep‐24™ 5 G instrument (MP Biomedicals) for 40 s at a setting of 6.0. After this initial step, the remaining protocol was followed as described by the supplier.

### 2.4 DNA Metabarcoding Library Preparation and Sequencing

Bacterial library was prepared by using a fragment of the 16S RNA gene (~300‐330 bp) that was amplified with 515F‐Y (5′‐GTGYCAGCMGCCGCGGTAA‐3′) and 806RB (5′‐GGACTACNVGGGTWTCTAAT‐3′) primers (Apprill et al. 2015; Parada et al. 2016; Vierna et al. 2017). These primers also included Illumina sequencing primer sequences attached to their 5′ ends. In the first amplification step, PCRs were carried out in a final volume of 12.5 μL, containing 1.25 μL of DNA as template, 0.5 μM of the primers, 3.13 μL of Supreme NZYTaq 2x Green Master Mix (NZYTech), and ultrapure water up to 12.5 μL. The reaction mixture was incubated at an initial denaturation temperature of 95°C for 5 min, followed by 25 cycles of 95°C for 30 s, 46°C for 45 s, 72°C for 45 s, and a final extension step at 72°C for 7 min. The oligonucleotide indices required for multiplexing different libraries in the same sequencing pool were attached in a second amplification step, carried out in a final volume of 25 µL, containing 2.5 µL of PCR product from the first amplification step, 1 µM dual‐indexed primers, 6.5 µL of Supreme NZYTaq 2x Green Master Mix (NZYTech), and ultrapure water up to 25 µL. The reaction mixture was incubated as follows: an initial denaturation step at 95°C for 5 min, followed by 5 cycles of 95°C for 30 s, 60°C for 45 s, 72°C for 45 s, and a final extension step at 72°C for 7 min. A negative control without DNA (BPCR) was included in every PCR round to check for contamination during library preparation. The library size was verified by running the libraries on 3% agarose gels stained with GreenSafe (NZYTech) and imaging them under UV light. Then, libraries were purified using the Mag‐Bind RXNPure Plus magnetic beads (Omega Bio‐tek), following the instructions provided by the manufacturer. Finished libraries were pooled in equimolar amounts according to the results of a Qubit dsDNA HS Assay (Thermo Fisher Scientific) quantification. The pool was sequenced in a fraction (1/8) of a MiSeq PE300 flow cell (Illumina). Finally, the library pool was sequenced using an Illumina MiSeq PE300 sequencing platform (Illumina Inc, San Diego, CA, USA) by the AllGenetics & Biology S.L. company (A Coruña, Spain).

### 2.5 Sequencing Data Processing

Microbiome bioinformatics was performed with the software Qiime2 2024.2 (Bolyen et al. 2019). After receiving the demultiplexed paired‐end reads from the sequencing service, the Illumina adapters were removed with Cutadapt (Martin 2011) and the sequences were trimmed according to their per‐base quality scores in FastQC (https://www.bioinformatics.babraham.ac.uk/projects/fastqc/). Filtered reads were denoised using the DADA2 software (Callahan et al. 2016). The resultant ASVs (amplicon sequence variants) were afterwards taxonomically classified using the SILVA ribosomal RNA database, release 138 SSU (Quast et al. 2013), and a pre‐trained classifier for the 515F‐806R region through scikit‐learn 0.24.1 (Bokulich et al. 2018). Demultiplexed raw sequence files were submitted to the NCBI Sequence Read Archive repository (www.ncbi.nlm.nih.gov/sra) and are accessible through the BioProject accession PRJNA1207528.

### 2.6 Statistical Analyses

Multivariate statistical analyses were carried out with the “R” software for statistical computing and graphing, version 4.4.0 (R Core Team 2024) and the community ecology package “vegan” (version 2.6‐6.1) (Oksanen 2024). To avoid bias related to different sequencing depths, the number of reads per sample was rarefied to the lowest one (sample LD4, 17926 sequences). Rarefaction curves were generated to ensure that rarefied sequencing depth was sufficient to describe the bacterial diversity of the communities. To carry out with α‐diversity metrics, species richness, Shannon, Simpson's Reciprocal, and Faith's Phylogenetic Diversity indices were calculated at the ASV level and were tested for significant differences between communities from different plastispheres. Homogeneity of variances and normality assumptions were previously verified by Bartlett and Shapiro‐Wilk tests, respectively. A Bray‐Curtis dissimilarity matrix was constructed at the ASV level to analyze the compositional and structural differences between bacterial communities from each type of plastic (β‐diversity). The same dissimilarity matrix was used to perform a 2D Nonmetric multidimensional scaling analysis (NMDS), a hierarchical clustering, a perMANOVA with 999 permutations, and a post‐hoc pairwise comparison (“pairwise Adonis”, package developed by Martinez and Arbizu 2020). Relative frequencies of each ASV were used to perform indicator species analyses (ISA) with the package “indicspecies” (version 1.7.14) (Cáceres and Legendre 2009) to identify bacterial ASVs associated with a specific plastisphere. ISA analysis was also conducted at the genus and family levels.

### 2.7 Functional Predictions of Microbial Communities

The functional analysis of bacterial communities was predicted using the 16S rRNA ASV data analyzed with the software PICRUSt2 v.6.6.0 (Douglas et al. 2020). ASV representative sequences from the plastisphere or the nearby soil used as control were aligned to the reference phylogeny using HMMER (Eddy 2011), EPA‐NG (Barbera et al. 2019), and GAPPA (Czech et al. 2020). The hidden state prediction tool was used to obtain 16S copy numbers per genome and to predict the Enzyme Commission (EC) gene family abundances for each ASV (Louca and Doebeli 2018). Then, the read depth per ASV was normalized using the estimated 16S rRNA gene copy number, and subsequently used to obtain the predicted functional profile for each sample, based on the EC gene family abundances. Finally, MetaCyc pathway abundances were inferred using MinPath (Ye and Doak 2009) and the MetaCyc reactions database. Differences in bacterial functional profiling pathways between the plastisphere and bulk soil were assessed using STAMP (Parks et al. 2014), where two‐sided Welch's *t*‐tests were performed with Benjamini–Hochberg multiple testing correction (*q*‐value\0.05).

## 3 Results and Discussion

### 3.1 Sampling and Spectroscopic Analysis of Plastic Materials

Plastic materials used in this study were collected from an agricultural landfill site in El Ejido (Almería, Spain) (Figure S1). This region of south‐eastern Spain is the largest exponent of greenhouse agriculture in Europe, where the dumping of agricultural waste is a common practice, contrary to the sustainability that has been pursued during the last decades (Castillo‐Díaz et al. 2021). Recent studies have demonstrated that in this region, the intensive use of plastics during the last four decades correlates with increasing microplastic particles accumulated in seagrass soil (Dahl et al. 2021). Plastic samples collected for this study were half buried, which ensures interaction with soil microorganisms. The concept of plastisphere has been defined mainly for plastic accumulated in marine environments, but in a more specific description, the soil plastisphere can comprise soil that is in direct contact with the plastic (Rillig et al. 2024). The ATR‐FTIR analysis of the plastic samples used for the plastisphere study showed spectra with characteristic absorption bands of LDPE and PP (Rajandas et al. 2012). In the plastic material composed of PP polymer, absorption bands at 2956 cm^−^^1^ and 2875 cm^−^^1^ (CH_3_ asymmetric and symmetric stretches), 2921 cm^−^^1^ and 2840 cm^−^^1^ (CH_2_ asymmetrical and symmetrical stretching), 1377 cm^−^^1^ (CH_3_ umbrella mode), and 1100 cm^−^^1^ (C‐C stretching or possible C‐O stretching) were identified, which correspond to standard signals of PP‐type material (Figure S2). For the LDPE‐based material, the FTIR spectrum showed absorption bands at 2917 cm^−^^1^ (asymmetric C‐H stretch), 2852 cm^−^^1^ (CH_2_ symmetric C‐H stretch), 1377 cm^−^^1^ (umbrella mode), 1100 cm^−^^1^ (C‐C stretching or possible C‐O stretching), and 718 cm^−^^1^ (CH_2_ rock), which are characteristics of LDPE‐polymer (Figure S3).

### 3.2 Composition of Bacterial Communities From the Plastisphere of Agricultural Residues

Microorganisms from the PP or LDPE plastisphere and from bulk soil (plastic‐free control) were collected as described in the Material and methods section. DNA was isolated, and sequencing of the 16S rRNA fragment was performed with amplification of the V4 region, yielding a total of 2,021,868 raw reads. After the quality‐filtering and pre‐processing steps, 264,520 reads were finally obtained, from which a total of 3969 amplicon sequence variants (ASVs) were inferred (Table S1). According to the rarefaction curves, most of the bacterial diversity was captured for the samples (sequencing depth of 17,926 reads per sample) (Figure S4).

**Figure 1:**
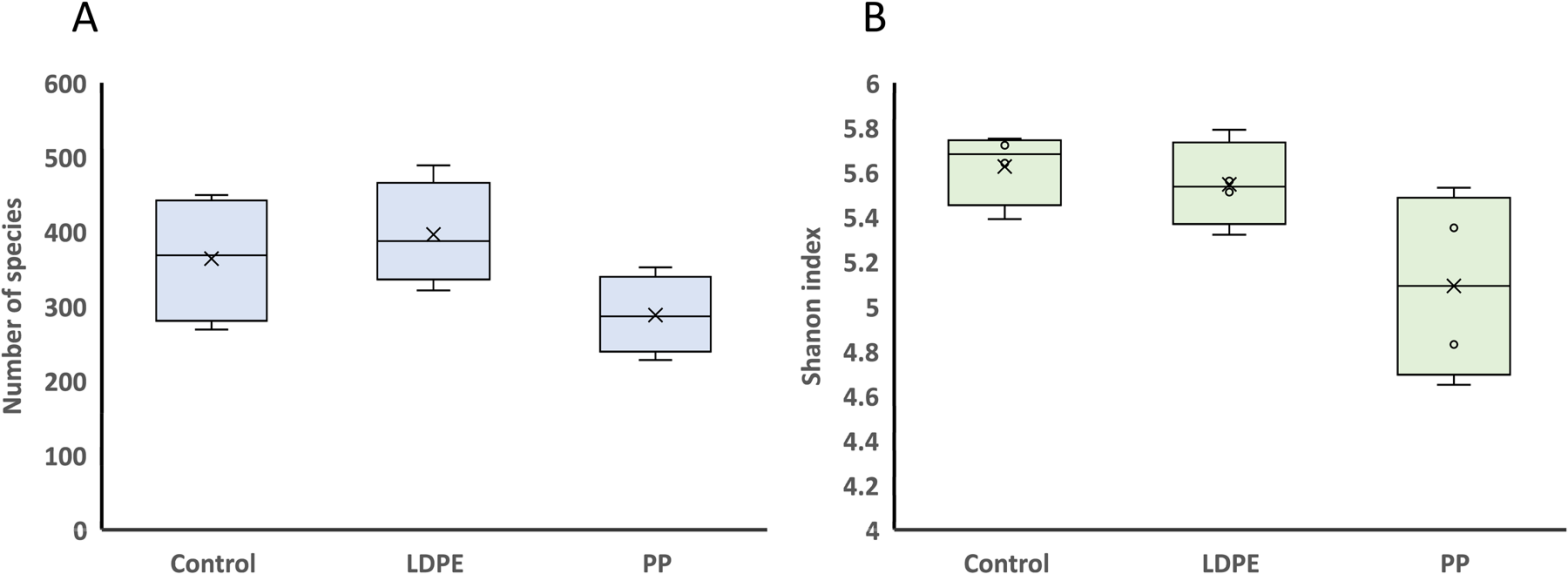
Richness (A) and diversity (B) of bacterial communities from LDPE and PP plastispheres and the nearby soil (bulk soil). The lower and upper hinges of the boxplots correspond to the 25th and 75th percentiles, respectively. The middle of the box denotes the median at 50th percentile. Whiskers denote 1.5 times the inter‐quartile range.

Bacterial richness and diversity in LDPE or PP plastisphere were estimated using different indices of α‐diversity (Figure 1). Although the number of species and the Shannon index in the PP plastisphere were lower than in the bulk soil control and in the LDPE plastisphere, these differences were not statistically significant. These results could suggest a reduced bacterial richness and diversity in the PP plastisphere, although additional studies will be required to confirm this observation. In addition, differences in evenness were not observed between plastispheres and plastic‐free soil. According to other studies, the plastisphere from agricultural soils containing plastic mulching or soils of abandoned landfill contaminated with polyethylene debris showed lower bacterial richness, diversity, and evenness than bulk soil (Bandopadhyay et al. 2020; Maclean et al. 2021; Sun et al. 2022; Zhang et al. 2019). In aquatic environments, lower richness and greater evenness on plastic debris regarding to the surrounding water have been described, although some exceptions have also been mentioned (Amaral‐Zettler et al. 2020). Differences in α‐diversity between LDPE and PP have not been described in previous studies, although this study suggests that LDPE plastisphere is a more complex niche than that of PP plastisphere.

**Figure 2:**
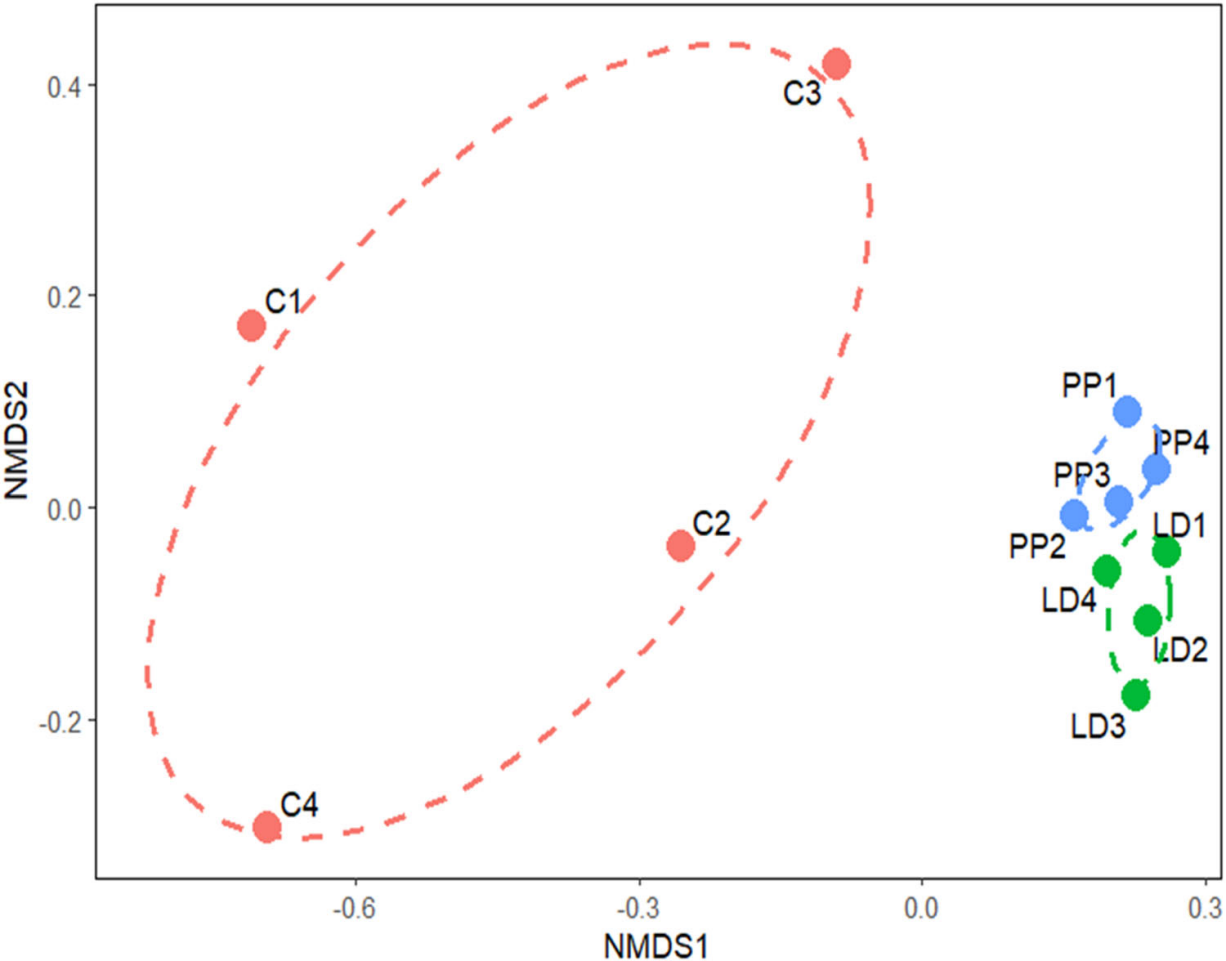
Beta diversity analysis of LDPE (LD1‐LD4) and PP (PP1‐PP4) plastispheres from an agricultural dump. Nonmetric dimensional scaling (NMDS) of samples. Plot ellipses show the 95% confidence regions for the locations of group centroids. Stress value was 0.042. C1–C4 represent samples from non‐contaminated soil (bulk soil).

The β‐diversity analysis, carried out by a Nonmetric multidimensional scaling based on Bray‐Curtis dissimilarity, showed that both LDPE and PP plastisphere bacterial communities clustered close, but separately from the control bulk soil (Figure 2). This result, in contrast to the absence of a significative difference in α‐diversity among samples (Figure 1), points to community turnover rather than diversity loss or gain, and it can be driven by environmental filtering where different taxa are selected, but the overall diversity remains stable. A microcosm experiment carried out with soil contaminated with polyethylene‐ or polylactic acid‐based microplastics showed that bacterial communities from the plastisphere are significantly distinct from the communities established in the bulk soil (Sun et al. 2022). Similar findings have been widely described in aquatic environments (McCormick et al. 2014; Zettler et al. 2013).

**Figure 3:**
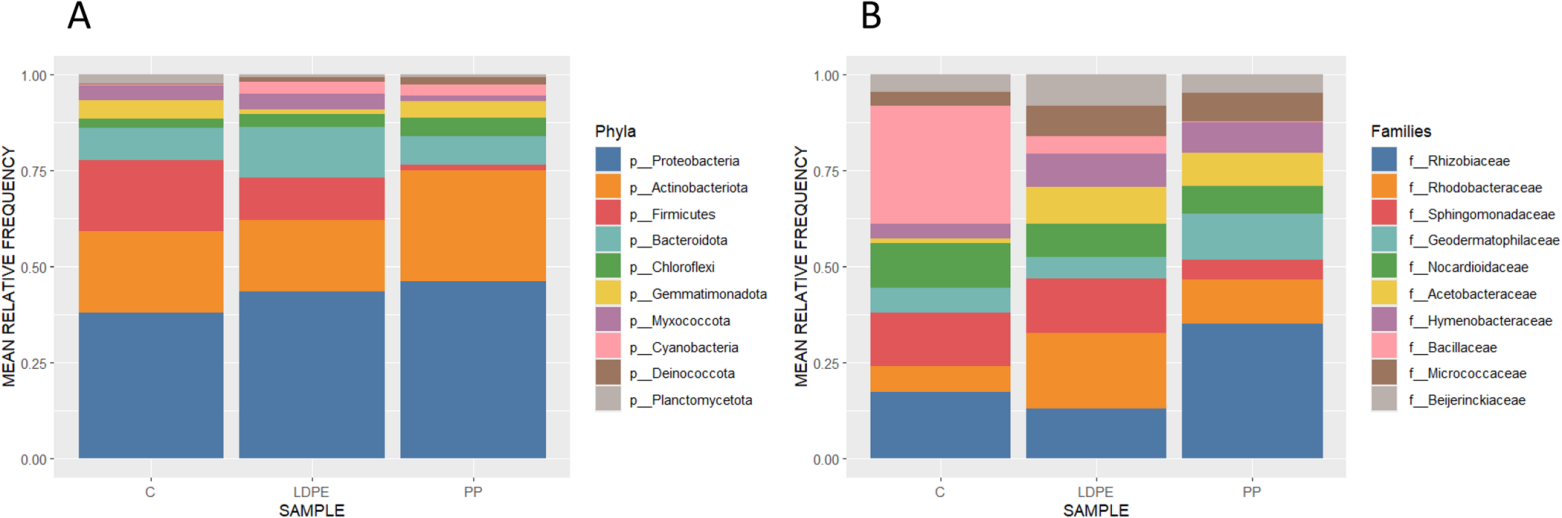
Taxonomic composition of the LDPE and PP plastisphere in an agricultural dump ecosystem. Data represent the mean of four replicates for the 10 most abundant microbial taxa at the phylum (A) and family (B) level. Samples were collected from the LDPE plastisphere, PP plastisphere or the nearby bulk soil in the absence of plastics (control, C).

The obtained ASVs were classified taxonomically using SILVA 132 database, yielding the bacterial taxonomy distribution at phylum and class levels for all samples (Figure 3). Considering the 10 most abundant phyla, Proteobacteria and Actinobacteriota were the dominant phyla in the different samples. This result has been described previously in different studies focused on soil microbiology (Delgado‐Vaquerizo et al. 2018; Zhang et al. 2019). In LDPE and PP plastispheres, the proportions of Cyanobacteria and Deinococcota were significantly higher than in the control soil, but in contrast, Planctomycetota showed a higher proportion in the bulk soil than in the plastisphere. Both the phylum Cyanobacteria and the class Deinococci have been described previously to be enriched in farmland plastic compared to the bulk soil (Bandopadhyay et al. 2020; Zhang et al. 2019). On the other hand, the proportions of Firmicutes and Myxococcota in the PP were significantly lower than those in the LDPE and the control soil (Figure 3A). However, Firmicutes is one of the phyla in which more plastic‐degrading bacteria have been previously reported, and it has been identified in abundance in PE plastisphere (Matjašič et al. 2021; Mohsen et al. 2022).

At the family level, Rhodobacteraceae, Acetobacteraceae, Hymenobacteraceae, Micrococcaceae and Beijerinckiaceae families showed a higher proportion of PP and LDPE plastispheres than the control soil. By contrast, Bacillaceae and Nocardioidaceae were predominant families dominant in the control soil with regard to the plastisphere (Figure 3B). Rhodobacteraceae is a dominant plastic‐degrading bacterial family, and it is one of the clades frequently associated with cyanobacteria (Halary et al. 2022; Mohsen et al. 2022). Members of the family Acetobacteraceae and the genus *Hymenobacter* have been found in the freshwater plastisphere (Bocci et al. 2024). In addition, an uncultured Acetobacteraceae was one of the major bacteria related to the biodegradation of polyethylene‐based microplastics (Taipale et al. 2023). Some bacterial plastic degraders have been described to inhabit the aquatic plastisphere, however, conditions in the ocean result in very slow rates of biodegradation (Amaral‐Zettler et al. 2020).

To establish possible associations between bacterial taxa and the plastisphere, an indicator species analysis (ISA) at the ASV level was performed. In this analysis, the species *Flavisolibacter ginsengisoli* and the family Comamonadaceae were identified as important indicators of the bacterial community from the LDPE plastisphere (Table 1). Comamonadaceae bacteria have been found to be enriched on PET microplastics, showing the ability to degrade this synthetic polymer (Wilkes et al. 2024). In the case of the PP plastisphere, *Blastococcus*, *Paracoccus*, *Arthrobacter* and *Kocuria* were found as indicator genera (Table 1). In a previous work, the *Blastococcus* and *Arthrobacter* genera reduced their relative abundance in soils exposed to LDPE microplastics (Ya et al. 2022).

**Table 1:** Indicator value (IndVal) for bacterial taxa at the ASV level of the LDPE or PP plastisphere from an agriculture dump.

Bacterial genus/species	IndVal*	*p* value
**LDPE**		
*Flavisolibacter ginsengisoli*	1.000	0.014
*Comamonadaceae*	1.000	0.014
**PP**		
*Arthrobacter*	1.000	0.004
*Blastococcus*	0.902	0.014
*Paracoccus*	0.894	0.011
*Arthrobacter*	0.866	0.040
*Arthrobacter*	0.838	0.031
*Paracoccus*	0.796	0.029
*Blastococcus*	0.796	0.006
*Kocuria*	0.762	0.037
*Blastococcus*	0.747	0.050

* IndVal is defined as an Indicator Value index to measure the association between a species and a site group.

### 3.3 Functional Analysis of Bacterial Communities From Agricultural Soil Plastisphere

**Figure 4:**
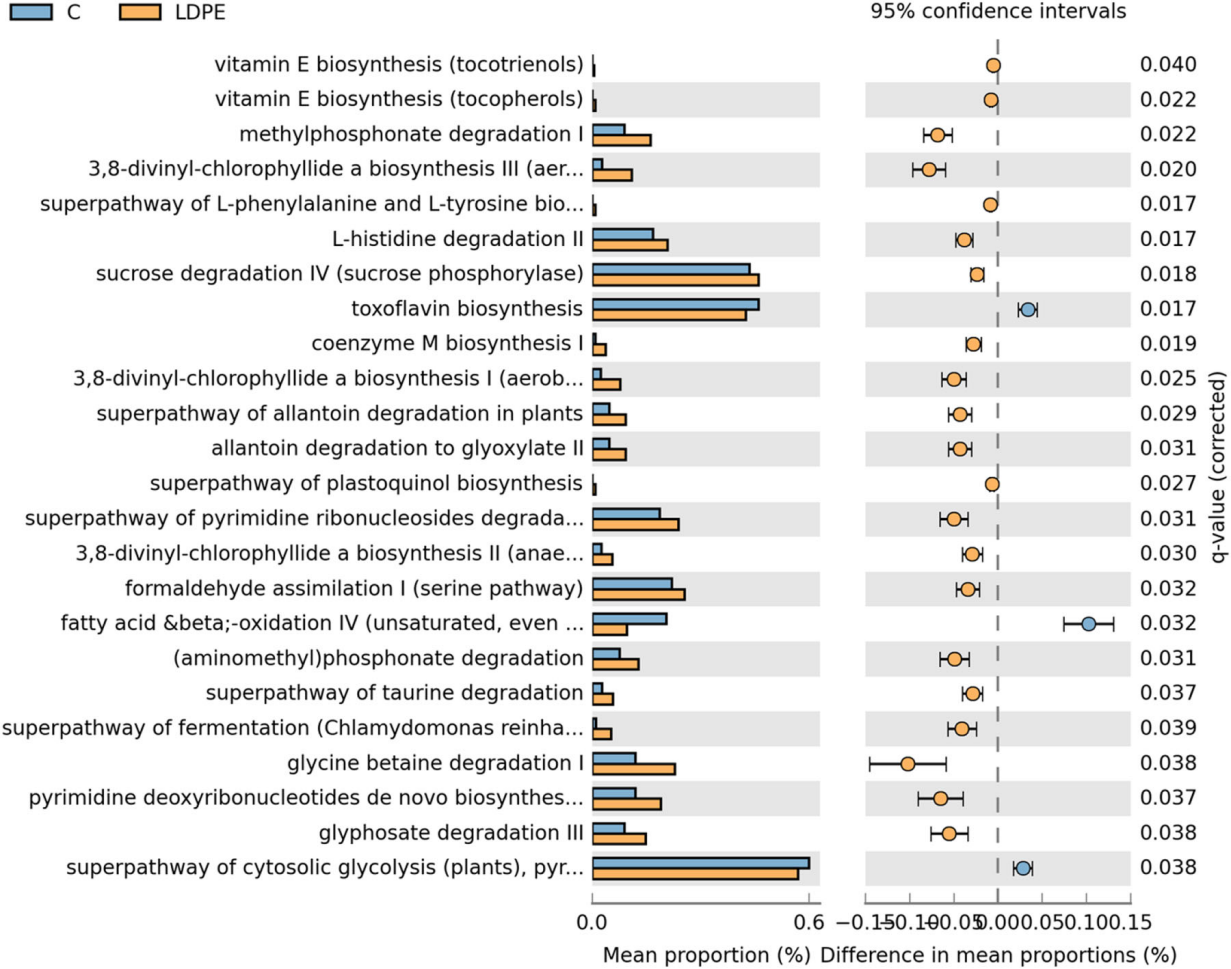
Comparison of the metagenome prediction pathways in LDPE plastisphere and bulk soil. The analysis was performed with the PICRUSt2 software and the MetaCyc database. All the pathways showed significant differences (corrected *p*‐value < 0.05) between the LDPE (orange) plastisphere and control soil (blue). A multiple test correction by the Benjamini‐Hochberg procedure was applied.

To elucidate the metabolic potential of LDPE and PP plastispheres, a functional predictive analysis using the 16S rRNA gene sequences obtained from the microbial populations of the LDPE or PP plastisphere and plastic‐free soil bulk soil was carried out. This functional study has been performed using PICRUSt2 software and the MetaCyc pathways database. The analysis of LDPE plastisphere vs. bulk soil showed that most of the differences corresponded to metabolic pathways over‐represented in LDPE (21), while only 3 routes were down‐represented in the plastisphere (Figure 4). Nine of the over‐represented pathways belong to biosynthetic processes, compared to 11 that correspond to catabolic pathways. Many of the biosynthetic pathways over‐represented in LDPE correspond to phototrophs, and these included metabolic pathways for plastoquinol, vitamin E, and 3,8‐divinyl‐chlorophyllide *a* (Figure 4). The electron carrier plastoquinol and the antioxidant vitamin E are exclusively synthesized by the photoautotrophic phylum Cyanobacteria. These two compounds are lipid‐soluble prenylquinones derived from homogentisate, an intermediate in the catabolism of phenylalanine and tyrosine. The biosynthetic pathway for these aromatic amino acids was also over‐represented in LDPE (Figure 4). The over‐representation of these biosynthetic pathways in LDPE agrees with the elevated proportion of the photoautotrophic phylum Cyanobacteria found in the plastisphere in comparison to the bulk soil (Figure 3A). 3,8‐divinyl‐chlorophyllide *a* is the precursor of bacteriochlorophyllides involved in anoxygenic photosynthesis in chlorophototrophic bacteria. The family Rhodobacteraceae, which includes chlorophototrophs, showed a higher proportion in the plastisphere than in the control without plastic (Figure 3B). Photodegradation of plastic caused by exposure to sunlight facilitates the access of microorganisms to the polymer plastic and releases products that can support bacterial growth. This could explain the taxonomic and functional relevance of phototrophic microorganisms in shaping the LDPE plastisphere observed in this study. The importance of phototrophs in the bacterial communities developed in the plastisphere has also been highlighted in coastal environments (Amaral‐Zettler et al. 2020; Bairoliya et al. 2024). The high proportion of Cyanobacteria found in this study on LDPE could also suggest moisture retention by this type of plastic.

The LDPE plastisphere also showed over‐representation of the biosynthetic pathway of the coenzyme M, which is the precursor of the methyl‐coenzyme M. This compound is the substrate of the methyl‐coenzyme M reductase (MCR), an enzyme involved in methanogenesis and anaerobic methanotrophy (Sarno et al. 2024). In a previous study, the presence of polystyrene microplastics has been described to affect the methanogenic activity of microbial communities from sewer sediments due to the inhibition of the MCR (Li et al. 2025). Recently, variants of MCR have been described to participate in the anaerobic oxidation of higher alkanes (Musat et al. 2024). The structural analogy of alkanes with plastic polymers suggests that alkyl‐coenzyme M reductases could use LDPE polymers as substrate, contributing to their oxidation. Other metabolic pathways over‐represented specifically in LDPE included fermentation and glycolytic pathways. In LDPE, the metabolism of pyrimidine was also over‐represented in comparison to the plastic‐free control (Figure 4). In *Burkholderia* sp. HQL1813, exposure to polylactic microplastics perturbed different metabolic pathways, such as nucleotide metabolism (Li et al. 2023). Other pathways specifically over‐represented in LDPE were the degradation of glyphosate and aminomethylphosphonate. Glyphosate is a synthetic phosphonate compound widely used as a nonselective systemic herbicide in agriculture and other environments. Aminomethylphosphonate is the most frequently detected intermediate of glyphosate degradation, and it is used as a source of inorganic phosphate (Zhan et al. 2018). This finding suggests that microorganisms from the LDPE plastisphere may have been more exposed to glyphosate than the microbiome developed in PP or the plastic‐free soil.

**Figure 5:**
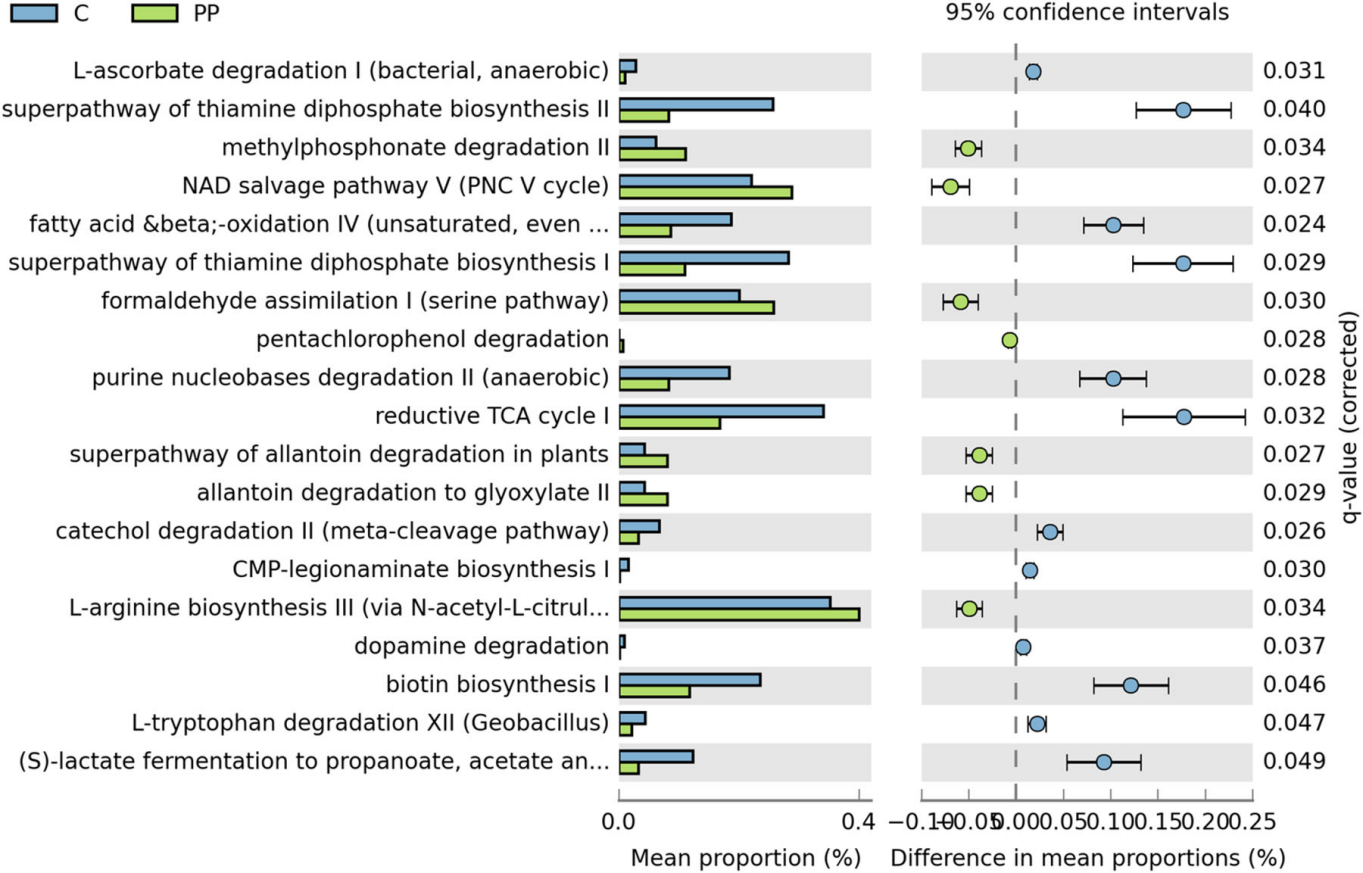
Comparison of the metagenome prediction pathways in PP plastisphere and bulk soil. The analysis was performed with the PICRUSt2 software and the MetaCyc database. All the pathways showed significant differences (corrected *p*‐value < 0.05) between the PP (green) plastisphere and control soil (blue). A multiple test correction by the Benjamini‐Hochberg procedure was applied.

In the analysis PP vs bulk soil, 19 metabolic pathways presented significant differences, of which 7 were over‐represented in PP and 12 were down‐represented in this plastisphere (Figure 5). Among pathways specifically over‐represented in PP, the biosynthetic pathway for l‐arginine, the pentachlorophenol degradation route, and the NAD salvage pathway were identified (Figure 5). Pentachlorophenol is a toxic organochlorine compound used predominantly as a wood preservative, but it has also been used as a pesticide. This phenolic compound has been applied in different industries, including food packaging and agriculture (Emenike et al. 2024). The enrichment of bacteria containing genes for the degradation of pentachlorophenol in PP suggests that this plastisphere has been exposed to this compound to a greater extent than LDPE or the bulk soil.

In the comparison of the results obtained for LDPE and PP, only the catabolic pathways for methylphosphonate, formaldehyde, and allantoin were shared (Figures 4, 5). In *Burkholderia* sp. HQL1813, the decomposition of methylphosphonate to methane has been described to be promoted by the presence of microplastics (Li et al. 2023). On the other hand, formaldehyde is widely used as a disinfectant and preservative, but it is also used in the industrial production of bio‐based rigid plastics (Ruan et al. 2024). Thus, the over‐representation of the formaldehyde assimilation pathway in the LDPE and PP plastisphere could be indicative of the utilization of this compound in agricultural practices. When the functional analysis was carried out considering a common plastisphere including both LDPE and PP in comparison to the bulk soil, the biosynthetic pathway of spirilloxanthin, the degradative route of protocathechuate and the metabolism of phospholipases were identified as over‐represented in the plastisphere (Figure S5). Spirilloxanthin is a carotenoid produced by some purple bacteria involved in excitation energy transfer in the photosynthetic process (Yakovlev and Taisova 2025). On the other hand, protocatechuate has been identified as a degradation product of dibutyl phthalate (DBP), which is commonly used as an additive in plastic production (Chen et al. 2025). *Arthrobacter* is one of the genera that exhibited a higher capacity for DBP degradation, and it contains the specific gene *pcaGH* responsible for protocatechuate degradation (Chen et al. 2025). Specifically, the genus *Arthrobacter* has been identified in this study as one of the indicators of the PP plastisphere. Therefore, microbial communities associated with the plastisphere might carry out the degradation of some plastic additives.

## 4 Conclusions

The plastisphere has been studied mainly in aquatic environments, while studies about the soil plastisphere are limited. Biodiversity studies focused on the soil plastisphere are revealing that bacterial communities colonizing this anthropogenic environment exhibit differences in comparison to natural soil. The β‐diversity analysis of the soil plastisphere from the agricultural landfill environment studied in this study has also shown differences among the bacterial communities developed on LDPE and PP synthetic plastics in comparison to the surrounding soil. Although the structure of the bacterial communities of both plastispheres exhibited a different pattern, they shared high proportions of phyla such as Cyanobacteria, Deinococcota, Rhodobacteraceae, and Acetobacteraceae, among others. The functional analysis of LDPE or PP plastispheres vs plastic‐free bulk soil revealed that in LDPE the differences consisted mainly in pathways over‐represented in the plastisphere, while in PP the number of over‐ and underrepresented routes was similar. This observation suggests that additional metabolic capacities are required to maintain the interactions in microbial communities in the LDPE plastisphere, but not in PP‐based plastic. The predictive metagenome analysis also suggested the degradation of plastic additives in both plastispheres, as well as the existence of degradation processes for specific herbicides in each plastisphere. The specificity of glyphosate in LDPE and pentachlorophenol in PP could be related to the differential attachment properties of these compounds to LDPE and PP. In agriculture, the use of herbicides that could act as hormonal disruptors has been highlighted as a major concern for human health.

On the other hand, the identification of bacterial phyla displaying capabilities to degrade synthetic plastics, the enrichment of biosynthetic pathways in phototrophs, and the probable existence of alkyl‐coenzyme M reductases that use LDPE polymers as substrate, may suggest the possibility that LDPE plastic is subject to both physical and biological degradation. As the functional analysis is not conclusive in assessing the potential of the plastispheres for LDPE and PP biodegradation, additional experimentation will be required. The expansion of studies on the plastisphere associated with different types of soil and environments constitutes the basis for determining the microbial communities that colonize these anthropic environments, which are considered a potential source of plastic‐degrading microorganisms (Bryant et al. 2016; McCormick et al. 2014; Zhang et al. 2019).

## Author Contributions

**Diego Becerra:** investigation, methodology. **Gema Rodríguez‐Caballero:** investigation, visualisation, software, data curation, formal analysis, writing – review and editing. **Frutos Carlos Marhuenda‐Egea:** methodology, resources. **Alfonso Olaya‐Abril:** methodology. **Conrado Moreno‐Vivián:** conceptualisation, funding acquisition, writing – review and editing. **Lara Paloma Sáez:** methodology, supervision, writing – review and editing. **Victor Manuel Luque‐Almagro:** writing – original draft, conceptualisation, writing – review and editing, formal analysis, visualisation. **María Dolores Roldán:** project administration, conceptualisation, funding acquisition, writing – review and editing, supervision.

## Ethics Statement

The authors have nothing to report.

## Conflicts of Interest

The authors declare no conflicts of interest.

## Supporting information


**Table S1:** Description of parameters for 16S rRNA metagenome sequencing. **Figure S1:** Sampling site and plastic materials used for the study of soil plastisphere. **Figure S2:** ATR–FTIR analysis of polypropylene (PP)–based materials collected from an agricultural dump. **Figure S3:** ATR–FTIR analysis of low‐density polyethylene (LDPE)–based materials collected from an agricultural dump. The typical absorption bands of polyethylene are indicated. **Figure S4:** Rarefaction curves calculated for the rarefied dataset. LD1–4: low‐density polyethylene replicates; PP1–4: polypropylene replicates; C1–4: control replicates (without plastics). **Figure S5:** Comparison of predicted metagenome pathways between the plastisphere (PP and LDPE) and the bulk soil. The analysis was performed using PICRUSt2 software and the MetaCyc database.

## Data Availability

The data supporting the findings of this study are openly available in NCBI Sequence Read Archive at https://www.ncbi.nlm.nih.gov/sra, with reference number PRJNA1207528.

## References

[mbo370121-bib-0067] Amaral‐Zettler, L. A. , E. R. Zettler , and T. J. Mincer . 2020. “Ecology of the Plastisphere.” Nature Reviews Microbiology 18, no. 3: 139–151. 10.1038/s41579-019-0308-0. 31937947

[mbo370121-bib-0001] Apprill, A. , S. Mcnally , R. Parsons , and L. Weber . 2015. “Minor Revision to V4 Region SSU rRNA 806R Gene Primer Greatly Increases Detection of SAR11 Bacterioplankton.” Aquatic Microbial Ecology 75, no. 2: 129–137. 10.3354/ame01753.

[mbo370121-bib-0002] Bairoliya, S. , J. Koh , Z. T. Cho , and B. Cao . 2024. “Phototrophs as the Central Components of the Plastisphere Microbiome in Coastal Environments.” Environment International 190: 108901. 10.1016/j.envint.2024.108901. 39079334

[mbo370121-bib-0003] Bandopadhyay, S. , J. E. Liquet y González , K. B. Henderson , M. B. Anunciado , D. G. Hayes , and J. M. DeBruyn . 2020. “Soil Microbial Communities Associated With Biodegradable Plastic Mulch Films.” Frontiers in Microbiology 11: 587074. 10.3389/fmicb.2020.587074. 33281783 PMC7691482

[mbo370121-bib-0004] Barbera, P. , A. M. Kozlov , L. Czech , et al. 2019. “EPA‐ng: Massively Parallel Evolutionary Placement of Genetic Sequences.” Systematic Biology 68, no. 2: 365–369. 10.1093/sysbio/syy054. 30165689 PMC6368480

[mbo370121-bib-0005] Blesa Marco, Z. E. , J. A. Sáez , F. J. Andreu‐Rodríguez , et al. 2024. “Effect of Abiotic Treatments on Agricultural Plastic Waste: Efficiency of the Degradation Processes.” Polymers 16, no. 3: 359. 10.3390/polym16030359. 38337248 PMC10857199

[mbo370121-bib-0006] Bocci, V. , S. Galafassi , C. Levantesi , et al. 2024. “Freshwater Plastisphere: A Review on Biodiversity, Risks, and Biodegradation Potential With Implications for the Aquatic Ecosystem Health.” Frontiers in Microbiology 15: 1395401. 10.3389/fmicb.2024.1395401. 38699475 PMC11064797

[mbo370121-bib-0007] Bokulich, N. A. , B. D. Kaehler , J. R. Rideout , et al. 2018. “Optimizing Taxonomic Classification of Marker‐Gene Amplicon Sequences With QIIME 2's q2‐feature‐classifier Plugin.” Microbiome 6, no. 1: 90. 10.1186/s40168-018-0470-z. 29773078 PMC5956843

[mbo370121-bib-0008] Bolyen, E. , J. R. Rideout , M. R. Dillon , et al. 2019. “Reproducible, Interactive, Scalable and Extensible Microbiome Data Science Using QIIME 2.” Nature Biotechnology 37, no. 8: 852–857. 10.1038/s41587-019-0209-9. PMC7015180 31341288

[mbo370121-bib-0009] Bryant, J. A. , T. M. Clemente , D. A. Viviani , et al. 2016. “Diversity and Activity of Communities Inhabiting Plastic Debris in the North Pacific Gyre.” mSystems 1, no. 3: e00024‐16. 10.1128/msystems.00024-16. 27822538 PMC5069773

[mbo370121-bib-0010] Cáceres, M. D. , and P. Legendre . 2009. “Associations Between Species and Groups of Sites: Indices and Statistical Inference.” Ecology 90, no. 12: 3566–3574. 20120823 10.1890/08-1823.1

[mbo370121-bib-0011] Callahan, B. J. , P. J. McMurdie , M. J. Rosen , A. W. Han , A. J. A. Johnson , and S. P. Holmes . 2016. “DADA2: High‐Resolution Sample Inference From Illumina Amplicon Data.” Nature Methods 13, no. 7: 581–583. 10.1038/nmeth.3869. 27214047 PMC4927377

[mbo370121-bib-0012] Castillo‐Díaz, F. J. , L. J. Belmonte‐Ureña , F. Camacho‐Ferre , and J. C. Tello‐Marquina . 2021. “The Management of Agriculture Plastic Waste in the Framework of Circular Economy. Case of the Almeria Greenhouse (Spain).” International Journal of Environmental Research and Public Health 18, no. 22: 12042. 10.3390/ijerph182212042. 34831794 PMC8625533

[mbo370121-bib-0013] Chae, Y. , and Y. J. An . 2018. “Current Research Trends on Plastic Pollution and Ecological Impacts on the Soil Ecosystem: A Review.” Environmental Pollution 240: 387–395. 10.1016/j.envpol.2018.05.008. 29753246

[mbo370121-bib-0014] Chen, F. , J. Chen , Y. Chen , et al. 2025. “Mechanistic Insight Into Degradation of Dibutyl Phthalate by Microorganism in Sediment‐Water Environment: Metabolic Pathway, Community Succession, Keystone Phylotypes and Functional Genes.” Environmental Pollution 371: 125932. 10.1016/j.envpol.2025.125932. 40020898

[mbo370121-bib-0015] Chung, J. , J. Yeon , H. J. Seong , et al. 2022. “Distinct Bacterial and Fungal Communities Colonizing Waste Plastic Films Buried for More Than 20 Years in Four Landfill Sites in Korea.” Journal of Microbiology and Biotechnology 32, no. 12: 1561–1572. 10.4014/jmb.2206.06021. 36453077 PMC9843814

[mbo370121-bib-0016] Crystal Thew, X. E. , S. C. Lo , R. N. Ramanan , B. T. Tey , N. D. Huy , and O. Chien Wei . 2024. “Enhancing Plastic Biodegradation Process: Strategies and Opportunities.” Critical Reviews in Biotechnology 44, no. 3: 477–494. 10.1080/07388551.2023.2170861. 36788704

[mbo370121-bib-0017] Czech, L. , P. Barbera , and A. Stamatakis . 2020. “Genesis and Gappa: Processing, Analyzing and Visualizing Phylogenetic (Placement) Data.” Bioinformatics 36, no. 10: 3263–3265. 10.1093/bioinformatics/btaa070. 32016344 PMC7214027

[mbo370121-bib-0018] Dahl, M. , S. Bergman , M. Björk , et al. 2021. “A Temporal Record of Microplastic Pollution in Mediterranean Seagrass Soils.” Environmental Pollution 273: 116451. 10.1016/j.envpol.2021.116451. 33486243

[mbo370121-bib-0051] De Souza Machado, A. A. , C. W. Lau , J. Till , et al. 2018. “Impacts of Microplastics on the Soil Biophysical Environment.” Environmental Science & Technology 52, no. 17: 9656–9665. 10.1021/acs.est.8b02212. 30053368 PMC6128618

[mbo370121-bib-0019] Delgado‐Baquerizo, M. , A. M. Oliverio , T. E. Brewer , et al. 2018. “A Global Atlas of the Dominant Bacteria Found in Soil | Enhanced Reader.” Science (New York, N.Y.) 359: 320–325. 10.1126/science.aap9516. 29348236

[mbo370121-bib-0020] Douglas, G. M. , V. J. Maffei , J. R. Zaneveld , et al. 2020. “PICRUSt2 for Prediction of Metagenome Functions.” Nature Biotechnology 38, no. Issue 6: 685–688. 10.1038/s41587-020-0548-6. PMC7365738 32483366

[mbo370121-bib-0021] Eddy, S. R. 2011. “Accelerated Profile HMM Searches.” PLoS Computational Biology 7, no. 10: e1002195. 10.1371/journal.pcbi.1002195. 22039361 PMC3197634

[mbo370121-bib-0022] Emenike, C. U. , Q. He , and K. Koushika . 2024. “Pentachlorophenol and Its Effect on Different Environmental Matrices: The Need for an Alternative Wood Preservative.” Sustainable Earth Reviews 7, no. 22. 10.1186/s42055-024-00090-x.

[mbo370121-bib-0074] European Commission . 2018. *European Strategy for Plastics in a Circular Economy* (COM/2018/028final).

[mbo370121-bib-0023] Halary, S. , S. Duperron , J. Demay , et al. 2022. “Metagenome‐Based Exploration of Bacterial Communities Associated With Cyanobacteria Strains Isolated From Thermal Muds.” Microorganisms 10, no. 12: 2337. 10.3390/microorganisms10122337. 36557590 PMC9785279

[mbo370121-bib-0024] Hofmann, T. , S. Ghoshal , N. Tufenkji , et al. 2023. “Plastics Can Be Used More Sustainably in Agriculture.” Communications Earth & Environment 4, no. 1: 332. 10.1038/s43247-023-00982-4.

[mbo370121-bib-0025] Jain, K. , H. Bhunia , and M. Sudhakara Reddy . 2018. “Degradation of Polypropylene–Poly‐L‐Lactide Blend by Bacteria Isolated From Compost.” Bioremediation Journal 22, no. 3–4: 73–90. 10.1080/10889868.2018.1516620.

[mbo370121-bib-0026] Li, J. , C. Yu , Z. Liu , Y. Wang , and F. Wang . 2023. “Microplastic Accelerate the Phosphorus‐Related Metabolism of Bacteria to Promote the Decomposition of Methylphosphonate to Methane.” Science of the Total Environment 858: 160020. 10.1016/j.scitotenv.2022.160020. 36356736

[mbo370121-bib-0027] Li, L. , T. Xiao , Z. He , and Q. Chen . 2025. “Concentration‐Dependent Effects of Polystyrene Microplastics on Methanogenic Activity and Microbial Community Shifts in Sewer Sediment.” Bioresource Technology 28: 132464. 10.1016/j.biortech.2025.132464. 40158865

[mbo370121-bib-0028] Louca, S. , and M. Doebeli . 2018. “Efficient Comparative Phylogenetics on Large Trees.” Bioinformatics 34, no. 6: 1053–1055. 10.1093/bioinformatics/btx701. 29091997

[mbo370121-bib-0029] Maclean, J. , S. Mayanna , L. G. Benning , et al. 2021. “The Terrestrial Plastisphere: Diversity and Polymer‐Colonizing Potential of Plastic‐Associated Microbial Communities in Soil.” Microorganisms 9, no. 9: 1876. 10.3390/microorganisms9091876. 34576771 PMC8465064

[mbo370121-bib-0030] Maity, S. , S. Banerjee , C. Biswas , R. Guchhait , A. Chatterjee , and K. Pramanick . 2021. “Functional Interplay Between Plastic Polymers and Microbes: A Comprehensive Review.” Biodegradation 32, no. 5: 487–510. 10.1007/s10532-021-09954-x. 34086181

[mbo370121-bib-0031] Martin, M. 2011. “Cutadapt Removes Adapter Sequences From High‐Throughput Sequencing Reads.” EMBnet Journal 17: 10–12. 10.14806/ej.17.1.200.

[mbo370121-bib-0032] Martinez , and P. Arbizu (2020). pairwiseAdonis: Pairwise multilevel comparison using adonis. R package version 0.4.

[mbo370121-bib-0033] Matjašič, T. , T. Simčič , N. Medvešček , O. Bajt , T. Dreo , and N. Mori . 2021. “Critical Evaluation of Biodegradation Studies on Synthetic Plastics Through a Systematic Literature Review.” Science of the Total Environment 752: 141959. 10.1016/j.scitotenv.2020.141959. 33207527

[mbo370121-bib-0034] McCormick, A. , T. J. Hoellein , S. A. Mason , J. Schluep , and J. J. Kelly . 2014. “Microplastic Is an Abundant and Distinct Microbial Habitat in an Urban River.” Environmental Science & Technology 48, no. 20: 11863–11871. 10.1021/es503610r. 25230146

[mbo370121-bib-0035] Moharir, R. V. , and S. Kumar . 2019. “Challenges Associated With Plastic Waste Disposal and Allied Microbial Routes for Its Effective Degradation: A Comprehensive Review.” Journal of Cleaner Production 208: 65–76. 10.1016/j.jclepro.2018.10.059.

[mbo370121-bib-0036] Mohsen, M. , C. Lin , H. I. Hamouda , A. M. Al‐Zayat , and H. Yang . 2022. “Plastic‐Associated Microbial Communities in Aquaculture Areas.” Frontiers in Marine Science 9: 895611. 10.3389/fmars.2022.895611.

[mbo370121-bib-0037] Musat, F. , K. U. Kjeldsen , A. E. Rotaru , S. C. Chen , and N. Musat . 2024. “Archaea Oxidizing Alkanes Through Alkyl‐Coenzyme M Reductases.” Current Opinion in Microbiology 79: 102486. 10.1016/j.mib.2024.102486. 38733792

[mbo370121-bib-0038] Narancic, T. , and K. E. O'connor . 2019. “Plastic Waste as a Global Challenge: Are Biodegradable Plastics the Answer to the Plastic Waste Problem?” Microbiology (Reading, England) 165, no. 2: 129–137. 10.1099/mic.0.000749. 30497540

[mbo370121-bib-0039] Oksanen, J. (2024). *Package “vegan” Title Community Ecology Package*. https://github.com/vegandevs/vegan.

[mbo370121-bib-0040] Parada, A. E. , D. M. Needham , and J. A. Fuhrman . 2016. “Every Base Matters: Assessing Small Subunit Rrna Primers for Marine Microbiomes With Mock Communities, Time Series and Global Field Samples.” Environmental Microbiology 18, no. 5: 1403–1414. 10.1111/1462-2920.13023. 26271760

[mbo370121-bib-0041] Parks, D. H. , G. W. Tyson , P. Hugenholtz , and R. G. Beiko . 2014. “Stamp: Statistical Analysis of Taxonomic and Functional Profiles.” Bioinformatics 30, no. 21: 3123–3124. 10.1093/bioinformatics/btu494. 25061070 PMC4609014

[mbo370121-bib-0042] Petersen, F. , and J. A. Hubbart . 2021. “The Occurrence and Transport of Microplastics: The State of the Science.” Science of the Total Environment 758: 143936. 10.1016/j.scitotenv.2020.143936. 33333307

[mbo370121-bib-0043] Quast, C. , E. Pruesse , P. Yilmaz , et al. 2012. “The Silva Ribosomal RNA Gene Database Project: Improved Data Processing and Web‐Based Tools.” Nucleic Acids Research 41, no. D1: D590–D596. 10.1093/nar/gks1219. 23193283 PMC3531112

[mbo370121-bib-0072] R Core Team . 2024. R: A Language and Environment for Statistical Computing. R Foundation for Statistical Computing.

[mbo370121-bib-0044] Rajandas, H. , S. Parimannan , K. Sathasivam , M. Ravichandran , and L. Su Yin . 2012. “A Novel FTIR‐ATR Spectroscopy Based Technique for the Estimation of Low‐Density Polyethylene Biodegradation.” Polymer Testing 31, no. 8: 1094–1099. 10.1016/j.polymertesting.2012.07.015.

[mbo370121-bib-0045] Rillig, M. C. , S. W. Kim , and Y. G. Zhu . 2024. “The Soil Plastisphere.” Nature Reviews Microbiology 22, no. 2: 64–74. 10.1038/s41579-023-00967-2. 37697003 PMC7615554

[mbo370121-bib-0046] Rong, Z. , Z. H. Ding , Y. H. Wu , and X. W. Xu . 2024. “Degradation of Low‐Density Polyethylene by the Bacterium Rhodococcus sp. C‐2 Isolated From Seawater.” Science of the Total Environment 907: 167993. 10.1016/j.scitotenv.2023.167993. 37866604

[mbo370121-bib-0047] Ruan, J. , J. Wang , C. Yang , W. Liu , F. He , and B. Zhong . 2024. “Biodegradation Enhancement of High Concentrations Formaldehyde Waste Gas and Verification of the Metabolic Mechanism.” Ecotoxicology and Environmental Safety 269: 115857. 10.1016/j.ecoenv.2023.115857. 38150844

[mbo370121-bib-0048] Saleem, M. , S. Yahya , S. A. Razzak , S. Khawaja , and A. Ali . 2023. “Shotgun Metagenomics and Computational Profiling of the Plastisphere Microbiome: Unveiling the Potential of Enzymatic Production and Plastic Degradation.” Archives of Microbiology 205, no. 11: 359. 10.1007/s00203-023-03701-x. 37884755

[mbo370121-bib-0049] Sarno, N. , E. Hyde , V. De Anda , and B. J. Baker . 2024. “Beyond Methane, New Frontiers in Anaerobic Microbial Hydrocarbon Utilizing Pathways.” Microbial Biotechnology 17, no. 6: e14508. 10.1111/1751-7915.14508. 38888492 PMC11184930

[mbo370121-bib-0050] Singh, P. , C. S. S. Lau , S. Y. Siah , K. O. Chua , and A. S. Y. Ting . 2024. “Microbial Degradation of Low‐Density Polyethylene, Polyethylene Terephthalate, and Polystyrene by Novel Isolates From Plastic‐Polluted Environment.” Archives of Microbiology 206, no. 4: 188. 10.1007/s00203-024-03895-8. 38519709

[mbo370121-bib-0052] Sun, Y. , J. Shi , X. Wang , C. Ding , and J. Wang . 2022. “Deciphering the Mechanisms Shaping the Plastisphere Microbiota in Soil.” mSystems 7, no. 4: e00352‐22. 10.1128/msystems.00352-22. 35880896 PMC9426546

[mbo370121-bib-0053] Taipale, S. J. , J. Vesamäki , P. Kautonen , et al. 2023. “Biodegradation of Microplastic in Freshwaters: A Long‐Lasting Process Affected by the Lake Microbiome | Enhanced Reader.” Environmental Microbiology 25: 2669–2680. 10.1111/1462-2920.16177. 36054230

[mbo370121-bib-0073] The MathWorks Inc . 2022. MATLAB version: 9.13.0 (R2022b). The MathWorks Inc. https://www.mathworks.com.

[mbo370121-bib-0054] Tiwari, N. , M. Bansal , D. Santhiya , and J. G. Sharma . 2022. “Insights into Microbial Diversity on Plastisphere by Multi‐Omics.” Archives of Microbiology 204, no. 4: 216. 10.1007/s00203-022-02806-z. 35316402

[mbo370121-bib-0055] Vierna, J. , J. Doña , A. Vizcaíno , D. Serrano , and R. Jovani . 2017. “PCR Cycles above Routine Numbers Do Not Compromise High‐Throughput DNA Barcoding Results.” Genome 60, no. 10: 868–873. 10.1139/gen-2017-0081. 28753409

[mbo370121-bib-0056] Wagner, M. , and J. Oehlmann . 2009. “Endocrine Disruptors in Bottled Mineral Water: Total Estrogenic Burden and Migration From Plastic Bottles.” Environmental Science and Pollution Research 16, no. 3: 278–286. 10.1007/s11356-009-0107-7. 19274472

[mbo370121-bib-0057] Wilkes, R. A. , N. Zhou , A. L. Carroll , et al. 2024. “Mechanisms of Polyethylene Terephthalate Pellet Fragmentation Into Nanoplastics and Assimilable Carbons by Wastewater Comamonas.” Environmental Science & Technology 58: 19338–19352. 10.1021/acs.est.4c06645. 39360733 PMC11526368

[mbo370121-bib-0058] Ya, H. , Y. Xing , T. Zhang , M. Lv , and B. Jiang . 2022. “Ldpe Microplastics Affect Soil Microbial Community and Form a Unique Plastisphere on Microplastics.” Applied Soil Ecology 180: 104623. 10.1016/j.apsoil.2022.104623.

[mbo370121-bib-0059] Yakovlev, A. G. , and A. S. Taisova . 2025. “Participation of Spirilloxanthin in Excitation Energy Transfer in Reaction Centers From Purple Bacteria Rhodospirillum Rubrum.” Photosynthesis Research 163, no. 1: 13. 10.1007/s11120-024-01126-1. 39870888

[mbo370121-bib-0060] Yang, S. S. , M. Q. Ding , L. He , et al. 2021. “Biodegradation of Polypropylene by Yellow Mealworms (*Tenebrio molitor*) and Superworms (Zophobas Atratus) via Gut‐Microbe‐Dependent Depolymerization.” Science of the Total Environment 756: 144087. 10.1016/j.scitotenv.2020.144087. 33280873

[mbo370121-bib-0061] Ye, Y. , and T. G. Doak . 2009. “A Parsimony Approach to Biological Pathway Reconstruction/Inference for Genomes and Metagenomes.” PLoS Computational Biology 5, no. 8: e1000465. 10.1371/journal.pcbi.1000465. 19680427 PMC2714467

[mbo370121-bib-0062] Yoshida, S. , K. Hiraga , T. Takehana , et al. 2016. “A Bacterium That Degrades and Assimilates Poly(Ethylene Terephthalate).” Science (New York, N.Y.) 351, no. 6278: 1196–1199. 10.1126/science.aad6359. 26965627

[mbo370121-bib-0063] Zettler, E. R. , T. J. Mincer , and L. A. Amaral‐Zettler . 2013. “Life in the ‘Plastisphere’: Microbial Communities on Plastic Marine Debris.” Environmental Science & Technology 47, no. 13: 7137–7146. 10.1021/es401288x. 23745679

[mbo370121-bib-0064] Zhan, H. , Y. Feng , X. Fan , and S. Chen . 2018. “Recent Advances in Glyphosate Biodegradation.” Applied Microbiology and Biotechnology 102, no. 12: 5033–5043. 10.1007/s00253-018-9035-0. 29705962

[mbo370121-bib-0065] Zhang, M. , Y. Zhao , X. Qin , et al. 2019. “Microplastics From Mulching Film Is a Distinct Habitat for Bacteria in Farmland Soil.” Science of the Total Environment 688: 470–478. 10.1016/j.scitotenv.2019.06.108. 31254812

[mbo370121-bib-0066] Zhang, Q. , R. Hu , J. Xie , X. Hu , Y. Guo , and Y. Fang . 2024. “Effects of Microplastics on Polycyclic Aromatic Hydrocarbons Migration in Baiyangdian Lake, Northern China: Concentrations, Sorption‐Desorption Behavior, and Multi‐Phase Exchange.” Environmental Pollution 366: 125408. 10.1016/J.ENVPOL.2024.125408. 39613180

