# Supplementary material for: Microbial Diversity of the Surface of Polypropylene and Low Density Polyethylene‐Based Materials (Plastisphere) From an Area Subjected to Intensive Agriculture
Source: Microbiologyopen. 2025 Dec 8;14(6):e70121. doi: 10.1002/mbo3.70121 (PMC12683173; doi:10.1002/mbo3.70121)
Supplement: Supplementary file 1 — Table S1: Description of parameters for 16S rRNA metagenome sequencing. Figure S1: Sampling site and plastic materials used for the study of soil plastisphere. Figure S2: ATR–FTIR analysis of polypropylene (PP)–based materials collected from an agricultural dump. Figure S3: ATR–FTIR analysis of low‐density polyethylene (LDPE)–based materials collected from an agricultural dump. The typical absorption bands of polyethylene are indicated. Figure S4: Rarefaction curves calculated for the rarefied dataset. LD1–4: low‐density polyethylene replicates; PP1–4: polypropylene replicates; C1–4: control replicates (without plastics). Figure S5: Comparison of predicted metagenome pathways between the plastisphere (PP and LDPE) and the bulk soil. The analysis was performed using PICRUSt2 software and the MetaCyc database. [file MBO3-14-e70121-s001.docx]

Table S1. Description parameters of the 16S rRNA metagenome sequencing.

| **Sample (#)** | **DNA**  **concentration in**  **extracts (ng/**μ**L)** | **DNA**  **concentration in libraries (ng/**μ**L)** | **Raw reads (R1 + R2) - 16S** | **Paired reads after filtering** | **ASVs**  **(*)** |
| --- | --- | --- | --- | --- | --- |
| Control 1 | >100 | 20.4 | 166 282 | 24 516 | 420 |
| Control 2 | 96.6 | 21.4 | 156 496 | 24 232 | 469 |
| Control 3 | 54.2 | 21.2 | 125 818 | 18 809 | 316 |
| Control 4 | >100 | 20.6 | 167 868 | 26 324 | 449 |
| LDPE 1 | 95.2 | 21.4 | 170 848 | 23 042 | 380 |
| LDPE 2 | >100 | 18.2 | 177 456 | 20 204 | 394 |
| LDPE 3 | 120 | 20.4 | 197 572 | 26 596 | 489 |
| LDPE 4 | 116 | 16.8 | 175 066 | 17 926 | 321 |
| PP 1 | 58.6 | 17.1 | 162 390 | 19 438 | 228 |
| PP 2 | 49 | 19 | 181 666 | 20 548 | 273 |
| PP 3 | 74 | 20 | 185 798 | 23 113 | 352 |
| PP 4 | 83.6 | 14.5 | 154 608 | 19 772 | 300 |

# Control 1-4: control replicates (without plastics). ; LDPE1-4: low-density polyethylene replicates; PP 1-4: polypropylene replicates.

* ASV: amplicon sequence variant

1

# El Ejido (Almería, Spain)

Latitude 36,7521/Longitude -2,8147


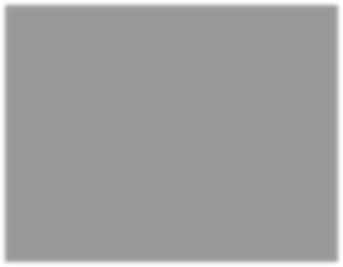

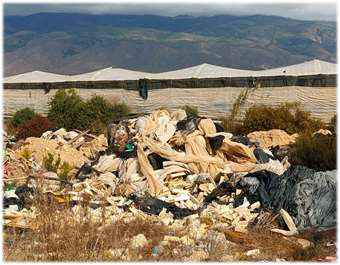

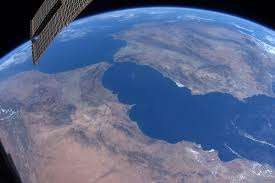

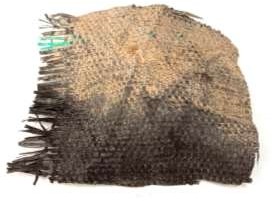

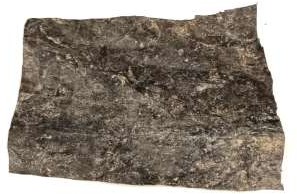


PP (Raffi)

Soil (control)

LDPE (plastic cover)

Figure S1. Sampling site and plastic material used for the study of soil plastisphere.

1100

1377

2840

2875

2956

2921


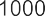

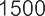

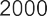

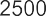

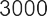


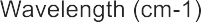


Figure S2. ATR-FTIR analysis of the PP-based materials collected from an agricultural dump. The characteristic absorption bands were identified and assigned based on known vibrational modes of the polypropylene structure.

718

1100

1377

2852

2917


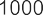

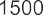

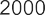

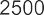

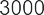

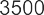


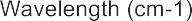


Figure S3. ATR-FTIR analysis of the LDPE-based materials collected from an agricultural dump. The typical absorption bands of polyethylene are indicated.


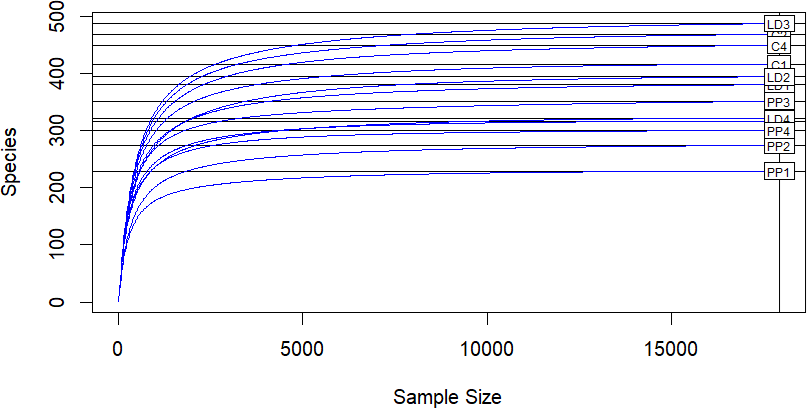


Figure S4. Rarefaction curves calculated for the rarefied dataset. LD1-4: low- density polyethylene replicates; PP1-4: polypropylene replicates; and C1-4: control replicates (without plastics).

5


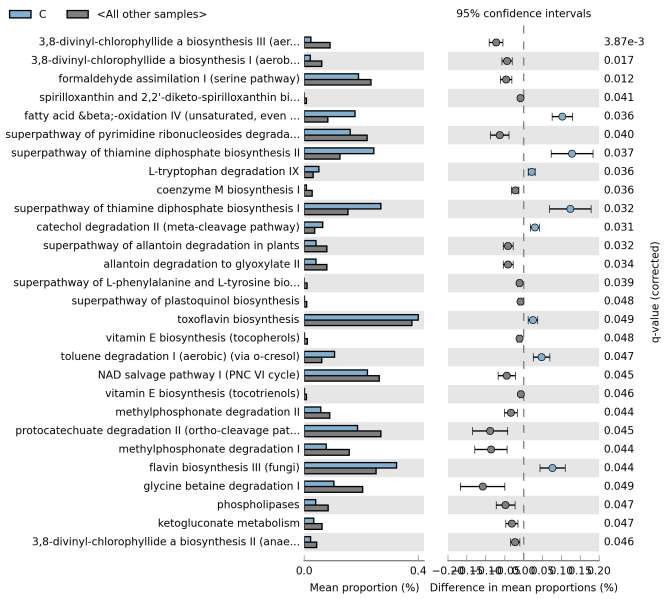


Figure S5. Comparison of the metagenome prediction pathways between the plastisphere (PP and LDPE) and

the bulk soil. The analys was performed with the PICRUSt2 software and the MetaCyc database. All the

showed pathways presented significative differences (correcte *p*-value < 0.05) among the plastisphere (grey) 6

and control soil (blue). A multiple test correction by the Benjamini-Hochberg procedure was applied.
